# Description of a New Labyrinthine Dehiscence: Horizontal Semicircular Canal Dehiscence at the Tympanic Segment of the Facial Nerve

**DOI:** 10.3389/fneur.2022.879149

**Published:** 2022-06-27

**Authors:** Gerard Gianoli, James Soileau, Bradley Shore

**Affiliations:** ^1^The Ear and Balance Institute, Covington, LA, United States; ^2^Diagnostic Imaging Services, New Orleans, LA, United States

**Keywords:** third mobile window, dehiscence, horizontal semicircular canal, facial nerve, vertigo, Tullio phenomenon, perilymphatic fistula

## Abstract

**Objective:**

This report is a case series of patients with findings suspicious for a labyrinthine dehiscence syndrome not previously described in the medical literature. We describe the clinical and test findings in 16 patients with CT findings suspicious for dehiscence of the ampullated end of the horizontal semicircular canal at the tympanic segment of the facial nerve.

**Study Design:**

Observational case series.

**Setting:**

Neurotology vestibular referral center.

**Patients:**

To be included in this study the patients were seen at our center in 2019 and had a high-resolution CT scan with a collimation of 0.6 mm. Patients who were identified as having findings suspicious for dehiscence of bone where the facial nerve crosses the ampullated end of the horizontal semicircular canal (HSC-FND) were identified and further analyzed.

**Interventions:**

Case series retrospective record review of patient symptoms, physical findings, audiometry, vestibular testing, and CT scans was performed. CT findings of other dehiscent sites were noted. A comparison to surgically treated perilymph fistula (PLF) patients of the same period was performed.

**Main Outcome Measures:**

History and physical exam were reviewed for auditory symptoms, vestibular symptoms, and exacerbating factors. and. Audiometry and vestibular testing were reviewed to determine which tests were most likely to be abnormal. CT scans were independently graded according to degree of suspicion for HSC-FND. Finally, patients with HSC-FND as the sole dehiscence identified were compared to those who had HSC-FND plus other dehiscent sites (HSC-FND+O) and to the group of surgically treated PLF patients.

**Results:**

Of 18 patients, 16 met inclusion criteria. Nine (56%) of those suspicious for HSC-FND had dehiscences in other parts of the labyrinth. Additional dehiscent sites included: six superior semicircular canal dehiscences (SSCD), two cochlear facial dehiscences and one cochlear carotid dehiscence. The most common auditory symptoms were autophony followed by tinnitus and aural fullness. The most common vestibular symptoms were pulsion sensation (feeling of being pushed to one side) followed by vertigo spells. The most common exacerbating factors for vertigo were straining, and sound. The most commonly abnormal vestibular test was nasal Valsalva testing, which was positive in all but one patient. Anamnesis and examination observations were similar in both groups, but the HSC-FND group were less likely to demonstrate a caloric weakness or an abnormal ECOG compared to the HSC-FND+O group. Of note, cVEMP was more often found to have lower thresholds in the HSC-FND group compared to the HSC-FND+O group. An example case is highlighted. Comparison to the PLF patients revealed statistically significant difference in the presenting symptoms of autophony, fullness and pulsion sensation. When comparing testing, HSC-FND patients were more likely to have an abnormal cVEMP and PLF patients were more likely to have asymmetric hearing. The incidence of bilateral disease was also more common among the HSC-FND patients than the PLF patients.

**Conclusions:**

A new labyrinthine dehiscence has been described to occur where the tympanic segment of the facial nerve crosses over the ampullated end of the horizontal semicircular canal. HSC-FND patients can present in a similar manner as HSC-FND+O patients with similar test findings except as mentioned above. The identification of one dehiscence such as SSCD does not preclude the presence of another dehiscence such as HSC-FND. HSC-FND could be the source of persistent symptoms post SSCD surgery as illustrated in the case presented. HSC-FND patients seem to identify themselves compared to PLF patients by a much more likely presenting symptoms of autophony, fullness, pulsion, abnormal cVEMP, bilaterality of disease, and symmetric hearing.

## Introduction

Superior Semicircular Canal Dehiscence (SSCD), first reported in 1998 (1), is a disorder defined as the absence of bone over the superior semicircular canal at the middle cranial fossa. The proposed pathophysiology is the Third Mobile Window Theory which posits abnormal compliance of the dehiscence (i.e., the third window) results in stimulation of the superior canal from egress of endolymph by changes in intracranial pressure or middle ear pressure. Its varying clinical presentations garnered it the nickname of the “Great Otologic Mimicker” (2). However, it has been most commonly characterized by the symptoms of Tullio's phenomenon, strain-induced vertigo/dizziness and autophony. Autophony to voice, heartbeat and eye movement are frequently reported with SSCD.

Since its identification it is being increasingly recognized that there are other bony dehiscences of the labyrinthine which can cause these same characteristic symptoms. Among these are posterior semicircular canal dehiscence (3), erosive processes into the horizontal semicircular canal, cochlear carotid dehiscence (4), cochlear facial dehiscence (5), enlarged vestibular aqueduct, X-linked gusher syndrome, cochlear-internal auditory canal defects (6), modiolar defects (7), and jugular bulb dehiscence into the vestibular aqueduct (8). Further, surgical repair or occlusion of SSCD has been shown to produce generally good outcomes for autophony and vestibular symptoms (9, 10). However, there are still some patients that have persistence of these symptoms in spite of what would seem to be successful surgery (11). This suggests an additional pathology or process in this subgroup of patients.

There are three elements to the diagnosis of Semicircular Canal Dehiscence Syndrome (SCD): (1) History consistent with SCD, namely Tullio's phenomenon, strain-induced vertigo and autophony, (2) Physiologic testing consistent with SCD (such as VEMP, ECOG, Tullio, Hennebert testing, etc.) and (3) CT imaging demonstrating a bony dehiscence of the superior semicircular canal (12). If the identifiable dehiscence is in another area of the otic capsule such as mentioned above, this is suspected to be the site of the pathology, i.e., Posterior Semicircular Canal Dehiscence, Cochlear Facial Dehiscence, etc. Many patients present with the first two elements of SCD syndrome but have no bony dehiscence of the labyrinth found on CT scan (13, 14).

In this paper we will describe a cohort of patients who meet criteria for labyrinthine dehiscence but have CT scan findings suspicious for a hitherto unreported bony dehiscence of the labyrinth. This dehiscence is located near the ampullated end of the horizontal semicircular canal bone at the crossover of the tympanic segment of the facial nerve (HSC-FND). We will present data on clinical presentation, audiovestibular test findings and imaging and compare to a group of patients with PLF. Our purpose is to help characterize what appears to be a newly identified labyrinthine dehiscence. We will also highlight a case of SSCD with concomitant HSC-FND who had persistent problems with autophony and vestibular symptoms despite appropriate surgical repair of the SSCD.

## Methods

The setting for this case series retrospective review is a tertiary Neurotology referral center for vestibular disorders that receives a high case load of SCD patient referrals. For the calendar year 2019, charts were retrospectively reviewed for the observation of HSC-FND on CT scan. These patients were referred to the primary author due to persistent symptoms of a third mobile window disorder. Further scrutiny of the CT scans identified 18 patients with a possible HSC-FND. The charts were analyzed for clinical symptoms, physical findings, audiometric testing, vestibular testing, and CT findings. Additionally, all patients had undergone MRI scanning with unremarkable findings.

Patients were excluded if CT was not available for review, or if CT slice thickness was inappropriately large. Patients were also excluded if another pathology (with the exception of other bony dehiscences) was identified that could explain the patient's symptomatology. Lastly, patients were excluded if they had not undergone audio-vestibular testing.

Historical information extracted from the charts included antecedent events, auditory symptoms of hearing loss, fluctuation, fullness, tinnitus, noise intolerance, hearing sensitivity, and autophony. When we use the term autophony, we refer to an enhanced perception of any bodily sound, including voice, heartbeat, and eye movement. Vestibular symptoms extracted included the presence of unsteadiness and vertigo. Exacerbating factors for vertigo–sound, straining and position–were also recorded. Physical findings recorded were Fukuda stepping test and the presence of spontaneous nystagmus during infrared video examination. Treatment and outcomes were recorded.

Comprehensive audiometry, electrocochleography and cervical vestibular evoked myogenic potentials (cVEMP) were analyzed. Results for caloric testing, Tullio testing, fistula testing, Valsalva testing and Platform Pressure Testing were analyzed.

cVEMP testing was performed with 500 Hz toneburst via insert electrodes. Surface electrodes on the sternocleidomastoid muscles during active muscle contraction recorded P1/N1 latencies and peak to peak amplitudes as well as thresholds. A threshold search was performed.

Tullio testing, fistula testing, and Valsalva testing was performed with VNG recording. Tullio testing was performed with the use of a portable audiometer. Sequential stimulation of each ear with 500 Hz pulsing tone at 105 db for 10 s each. Fistula testing was accomplished using a Bruening Otoscope with direct visualization of the tympanic membrane. Alternating positive and negative pressure was applied while looking for eye deflections synchronized with the pressure application over a thirty second time period. Valsalva testing was done using both glottic and nasal Valsalva over a thirty second period for each. Valsalva testing was defined as abnormal if the patient developed nystagmus and/or vertigo during the test. Platform pressure testing was accomplished on an Equitest sensory organization test number 5 with alternating positive, negative or no pressure applied to each ear sequentially. Abnormal results were noted for >50% increase in sway energy over baseline. If a patient was unable to maintain balance on sensory organization test number 5, then the Platform Pressure Test was deferred.

For the CT studies we used the standard application (inner ear high-resolution program) as suggested by the manufacturer. The parameters used with the Hitachi Supria (Hitachi HeathCare Tokyo, Japan) system were as follows: 120 kV, 125 mAs, pitch 0.562, 2.5 × 0.6 mm collimation, matrix 512 × 512; reconstruction using the bone algorithm to yield 0.6-mm-thick sections with an increment of 0.06 mm; field of view 8 cm; image length 5 cm; imaging time 20 s; reconstruction with extended CT scale.

The CT protocols were intended to achieve the highest spatial resolution. The Poschl projection was obtained perpendicular to the long axis of the petrous bone, at an angle of 45 degrees with the sagittal and coronal planes. The superior semicircular canal was seen as a ring in a single plane in the Poschl projection. The Stenvers projection was obtained parallel to the petrous apex and perpendicular to the Poschl plane. Specifically, for analyzing the scans for HSC-FND the coronal and Poschl images were reviewed.

These CT scans of these patients were analyzed by two Neurotologists and one Neuroradiologist for the degree of certainty of HSC-FND on a scale of 0-3. A “0” represented findings of no bony dehiscence in this region. Whereas, a “3” represented a high degree of certainty for a bony dehiscence being present at the region of the horizontal semicircular canal where the tympanic segment of the facial nerve crosses it laterally with at least two slices demonstrating dehiscence. The scores were averaged between the three reviewers. For the purpose of our study, an average score below 1.5 was considered to not have HSC-FND, while score ≥1.5 was considered to have HSC-FND. The scans were reviewed for concomitant bony dehiscences in other parts of the labyrinth (HSC-FND+O). Findings for HSC-FND patients were compared to HSC-FND+O patients.

The HSC-FND patients were compared to a group of seven surgically treated PLF patients from the same time period. The PLF group had no evidence of otic capsule dehiscence on CT scan and were successfully treated with surgical repair. Chi square analysis was applied to compare symptom presentation, vestibular testing, auditory testing and bilaterality of disease.

The procedures followed were in accordance with the ethical standards of the responsible committee on human experimentation and with the Helsinki Declaration. The Salus Institutional Review Board approved this study.

## Results

There were 18 patients identified in our chart review. Of these, one patient was excluded because the CT scan was not available for review. Another patient was excluded because they had not undergone vestibular testing. Consequently, there were 16 patients available for analysis.

Using our grading system, the presence of HSC-FND was noted to be bilateral in 13 cases and unilateral in 3 cases. The three reviewers were highly consistent with average score of 2.1, ranging from 1 to 3 (out of a possible 3) for all the areas analyzed. There was also highly consistent agreement on the scans that did not have an HSC-FND. The correlation coefficient comparing examiner 1 to examiner 2 was 0.61; for examiner 2 compared to examiner 3 it was 0.60; and for examiner 1 compared to examiner 3 it was 0.73.

Nine patients had findings of other dehiscent sites whereas seven had no other identifiable otic capsule dehiscences. The additional dehiscent sites included SSCD (six cases), Facial-Cochlear Dehiscence (FCD) (two cases) and one case of Cochlear Carotid Dehiscence (CCD). All patients had undergone MRI scan with no pathologic findings of note.

Of the 16 patients, 10 patients reported onset of symptoms immediately after trauma. Eight of these were after direct blunt head trauma. Two of these were subsequent to barotrauma–one during air travel and another with scuba diving.

Auditory and vestibular symptoms, as well as testing, are detailed in Table 1. The most common auditory symptoms included autophony (16, 100%), tinnitus (13, 81%), aural fullness (12, 75%) and noise intolerance (10, 63%). The most common vestibular symptoms were positional vertigo/dizziness (13, 81%), pulsion sensation (12, 75%), strain-induced vertigo/dizziness (12, 75%), non-positional rotary vertigo (11, 69%), unsteadiness (9, 56%) and Tullio phenomenon (8, 50%).

**Table 1:** Auditory symptoms, vestibular symptoms and testing for HSC-FND patients (*N* = 16) compared to PLF patients (*N* = 7).

**Findings**	**HSC-FND (*N* = 16)**	**PLF (*N* = 7)**	
Age	43 (range 17–63)	54 (36–69)	
Male:Female	9:7	4:3	
Trauma history	10 (63)^*^	5 (71)	*p* = 0.679
**Auditory symptoms**			
Autophony	16 (100)	0 (0)	*p* < 0.05
Tinnitus	13 (81)	6 (86)	*p* = 0.479
Aural fullness	12 (75)	2 (29)	*p* < 0.05
Noise intolerance	10 (63)	3 (43)	*p* = 0.382
Otalgia	7 (44)	3 (43)	*p* = 0.968
Hearing loss	5 (31)	4 (57)	*p* = 242
Fluctuation of hearing	5 (31)	2 (29)	*p* = 0.898
Hearing hypersensitive	3 (19)	0 (0)	*p* = 0.698
**Vestibular symptoms**			
Positional vertigo/Dizziness	13 (81)	5 (71)	*p* = 0.738
Pulsion sensation	12 (75)	1 (14)	*p* < 0.05
Strain-induced vertigo/Dizziness	12 (75)	5 (71)	*p* = 0.858
Rotary vertigo (non-positional)	11 (69)	5 (71)	*p* = 0.898
Unsteadiness	9 (56)	6 (86)	*p* = 0.172
Tullio phenomenon	8 (50)	3 (43)	*p* = 0.752
**Testing**			
Valsalva (Nasal or Glottic) Test	15 (94)	6(86)	*p* = 0.529
Fistula test	14 (88)	7 (100)	*p* = 0.743
Platform pressure test	9 (82)^**^	5 (71)	*p* = 0.605
Tullio testing	11 (69)	2 (29)	*p* = 0.074
ECOG	6 (40)++	1 (14)	*p* = 0.228
Caloric testing (UW > 25%)	5 (31)	4 (57)	*p* = 0.242
cVEMP	10(63)	1 (14)	*p* < 0.05
Asymmetric HL	4 (25)	6 (86)	*p* < 0.05
Bilateral disease	13 (81)	2 (29)	*p* < 0.05

* *Eight cases of direct head trauma and 2 cases of barotrauma*.

** *N = 11, Four patients could not stand on SOT #5 and one patient surpassed the weight limit for testing. Consequently, a total of 5 patients could not complete Platform Pressure Testing*.

++ *N = 15, One patient could not be evaluated because of no measurable SP wave*.

Physical exam demonstrated evidence of spontaneous nystagmus with infrared video exam in seven (44%) patients. An abnormal Fukuda test was found in 11 (69%) patients, but one patient (6%) was unable to stand to perform the Fukuda test. Three patients (19%) had no evidence of nystagmus and a normal Fukuda test. Audiometry was normal in 12 patients (75%). There were two patients (13%) who had bilateral asymmetric sensorineural hearing loss, one (13%) with a unilateral sensorineural hearing loss and one (13%) with symmetric sensorineural scores but had a unilateral conductive gap.

The most common vestibular test abnormalities were nasal and/or glottic Valsalva test (15, 94%), fistula test (14, 88%), Platform Pressure Test (9, 82%) and Tullio test (11, 69%). It should be noted that five patients could not perform the Platform Pressure test because four could not stand on Sensory Organization Test #5 and one was over the weight limit for Platform Testing.

Comparison of HSC-FND to HSC-FND+O demonstrated similar historical and physical exam qualities. There were a few differences in the HSC-FND and HSC-FND+O groups when it came to testing. Most notable was a higher incidence of ECOG abnormalities (SP/AP ratio > 0.40) in the HSC-FND+O group (56%) vs. the HSC-FND group (17%). Unilateral caloric weakness was also more common among the HSC-FND+O group (44%) vs. HSC-FND (17%). However, reduced cVEMP thresholds were more common among the HSC-FND group (83% were ≤85 db) compared to the HSC-FND+O group (56% were ≤85 db); and abnormal Valsalva testing was similar in both groups−100% for HSC-FND vs. 89% for HSC-FND+O. None of these outcomes were statistically significant. However, this may be due to the small total number of cases involved in this study.

There were 6 of 16 with no nystagmus on Tullio testing (5 HSC-FND+O and 1 HSC-FND) although some had symptoms but no nystagmus. There were 6 of 16 with horizontal nystagmus (1 HSC-FND+O and 5 HSC-FND). There were 4 of 16 with vertical/torsional nystagmus (3 HSC-FND+O and 1 HSC-FND). Another way to view this is that among those with HSC-FND (*N* = 7), on Tullio testing 5 had horizontal nystagmus, one had vertical/torsional nystagmus and one had no nystagmus. Whereas, among HSC-FND+O (*N* = 9) patients, Tullio testing resulted in 3 with vertical/torsional nystagmus, one with horizontal nystagmus and 5 with no nystagmus.

The fistula testing was more inclined to demonstrated horizontal phase-locked eye movement regardless of the group. Among HSC-FND patients, horizontal eye movement was noted in 6 of 7 patients with 1 of 7 demonstrating no eye movement. Among HSC-FND+O patients, 5 of 9 demonstrated horizontal phase-locked eye movement, 3 of 9 demonstrated vertical/torsional eye movement and 1 of 9 demonstrated no eye movement.

Comparison to the surgically treated PLF patients as detailed in Table 1, demonstrated statistically significant differences for the symptoms of autophony (100 vs. 0%), aural fullness (75 vs. 29%), pulsion sensation (75 vs. 14%). Testing demonstrated significant differences in cVEMP (63 vs. 14%), and asymmetric hearing loss (25 vs. 86%). Lastly, PLF patients were less likely to have bilateral disease (29%) compared to HSC-FND patients (81%).

All 16 HSC-FND patients were given medical management which consisted of dietary restrictions (low salt and caffeine avoidance), physical restrictions (avoidance of abdominal/thoracic straining) and either diuretics or a carbonic anhydrase inhibitor. One patient was lost to follow up. Of the remaining 14 patients, 10 (71%) had resolution of their vestibular symptoms with medical management. Four underwent surgical reinforcement of the suspected area, inclusive of the oval window along with round window reinforcement. One had sequential bilateral surgical repair, and one had unilateral repair–both with complete resolution of their vestibular symptoms. The third patient who underwent surgery completed the first of planned bilateral sequential repairs noting significant improvement in their symptoms at present. At the time of this writing, it is uncertain whether the contralateral procedure will be required. The fourth case is detailed below:

### Case Report

A 36-year-old female experienced onset of autophony, Tullio phenomenon and strain-induced vertigo after a head injury seven years prior. She was evaluated and treated at an outside facility for SSCD identified on CT scan, supported by an abnormal cVEMP result with a 55 db threshold. A middle fossa craniotomy with SSCD occlusion was performed. Postoperatively the patient noted no change in symptoms, with persistent autophony, Tullio's and strain-induced vertigo. The following year she was referred to our facility for evaluation. Audiometry was normal. cVEMP testing demonstrated thresholds improved to 85 db. VNG demonstrated balanced caloric testing. Tullio testing and Valsalva testing were abnormal, producing nystagmus and the symptoms of vertigo. Fistula testing produced synchronous eye movement with positive and negative pressure application to the ear canal. vHIT testing revealed reduced function in the superior canal that had been surgically occluded, suggesting the occlusion was successful. An MRI scan demonstrated a filling defect of the superior canal, again suggesting successful surgical occlusion of the superior canal. CT scan demonstrated HSC-FND as well as the previously noted SSCD and middle fossa craniotomy postoperative changes. She was referred back to her referring physician with therapeutic recommendations. She underwent window reinforcement by the referring surgeon that included the HSC-FND, oval and round window reinforcement. This resulted in complete resolution of her vestibular symptoms and improvement in her autophony as of her last communication which was approximately 6 months postoperative.

## Discussion

Most commonly, dehiscence or fistula of the horizontal semicircular canal has been identified to occur secondary to erosion from cholesteatoma (15). While this has been extensively reported in the medical literature, dehiscence of the horizontal semicircular canal not caused by some erosive process is not. Only two case reports of horizontal semicircular canal dehiscence were identified in our review of PubMed.

In 2007, Bassim et al. (16) reported a case of bony dehiscence of the horizontal semicircular canal in a patient being evaluated for cochlear implantation. The dehiscence was located throughout the entire apex of the arch of the horizontal semicircular canal and included more than half of the semicircular canal. The patient had a history of radiation therapy for lymphoma of the palate, but the authors did not suspect this as the cause of the dehiscence because of its unilaterality. The contralateral side had normal bony coverage. No vestibular symptoms and no physiologic testing were reported in this paper.

In 2010, Zhang et al. (17) reported a case of a 76-year-old with vertigo, Tullio phenomenon and autophony. The patient exhibited nystagmus with sound application to the right ear. A CT scan demonstrated a 2.0 mm bony defect of the right horizontal semicircular canal. They recommended consideration of dehiscence of the horizontal semicircular canal be included in the differential diagnosis of sound- or pressure-induced vestibular symptoms. We could not find in PubMed any reports of horizontal semicircular canal dehiscence located at the tympanic segment of the facial nerve.

As noted in the introduction, the proposed pathophysiology of SSCD is due to the abnormal compliance of the inner system due to the dehiscence of the superior canal bone at the temporal lobe dura (1). This renders the superior canal (as well as the otolithic organs) susceptible to abnormal pressure changes from the middle ear and from the temporal lobe dura. In HSC-FND, the defect is qualitatively different from SSCD. It is a dehiscence that is covered by the facial nerve, which by itself should provoke no pressure changes such as seen with dural pressure transmission in SSCD. In our patient population, 63% developed symptoms after head trauma. This raises the question as to whether trauma played a role. There were no fractures identified on CT scan or intraoperative. We propose that the pressure wave from the head trauma could have caused a shift in the facial nerve or an increased compliance in this area resulting in the third mobile window symptoms in these patients.

The only clinical symptom seen in every patient of our case series was autophony. This is in striking contrast to the PLF patient group were there were no complaints of autophony. Beyond this, the most common auditory symptoms were tinnitus, aural fullness, and noise intolerance. Vestibular symptoms of rotary vertigo and disequilibrium were provoked by positional changes, straining and sound (Tullio's phenomenon). While most patients had normal or symmetric hearing, cVEMP testing and vestibular testing was frequently abnormal. Most notable was Valsalva testing with all but one patient having an abnormal Valsalva test. The fistula test was abnormal in all but two patients and the Tullio test was abnormal in almost two thirds of patients. The Platform Pressure Test was abnormal in 82% of the patients who completed the test. However, there were five patients who could not perform Platform Pressure Testing–four because they could not maintain their balance for baseline testing (SOT#5) and one because they were beyond the weight limit capacity for the platform. The above clinical profile of symptoms and test findings are suggestive of the SCD patients we have seen over the last 22 years and that have been reported in the literature.

A surprising number of patients (75%) reported pulsion sensation–a rocking/swaying feeling or feeling like being pushed to one side–suggesting abnormal otolithic stimulation, compared to only 14% (*p* < 0.05) among the PLF patients. The attribution to central pathology of the pulsion/rocking sensation has been longstanding and without any significant studies to back this up. More recently, these symptoms have been attributed to otolithic stimulation (18). Intuitively, this makes more sense, since isolated otolithic stimulation would result in a pulsion sensation rather than a rotary sensation; and this is something we have observed in our patient population as well. Given that the otoliths are located anatomically near this dehiscence, pulse waves from vascular pressure changes could theoretically be the cause for the rocking sensation experienced. In fact, some of the patients reported the rocking to be in concert with their heartbeat.

Clinical findings of HSC-FND compared with HSC-FND+O patients did not differ statistically. In fact, the HSC-FND group was slightly more likely to have lowered thresholds on cVEMP and abnormal Valsalva test results than the HSC-FND+O group. Compared to the PLF group, only 14% had an abnormal cVEMP compared to 63% among the HSC-FND group (*p* < 0.05). This further supports the theory that HSC-FND is an alternative otic capsule dehiscent instrumental in creating dehiscent symptoms/test findings.

What was notable in both the HSC-FND group and to a lesser extent, the HSC-FND+O group was the presence of horizontal nystagmus on Tullio testing and horizontal phase-locked eye movement on fistula testing. Overall, on Tullio testing 6 patients had horizontal nystagmus, 4 had vertical/torsional nystagmus, and 6 had no nystagmus. Three of the 4 patients with vertical/torsional nystagmus were among the HSC-FND+O group that included SSCD and other dehiscences. On fistula testing, 11 patients had horizontal phase-locked eye movements, 3 had vertical/torsional phase-locked eye movements and 2 had no eye movement. All three who had vertical/torsional eye movements were in the HSC-FND+O group and had SSCD. The finding of horizontal nystagmus on Tullio testing and horizontal phase-locked eye movement with fistula testing would implicate horizontal semicircular canal stimulation. This finding would seem to support HSC-FND as a cause for the patients' symptomatology.

The presence of similar symptoms in HSC-FND could explain what some authors have reported as oval window perilymphatic fistula. HSC-FND could result in labyrinthine fistula from the ampullated end of the horizontal semicircular canal, around the facial nerve canal and into the oval window niche. Theoretically, reinforcement of the oval window would potentially reinforce the HSC-FND deficient area resulting in cessation of symptoms.

**Figure 1:**
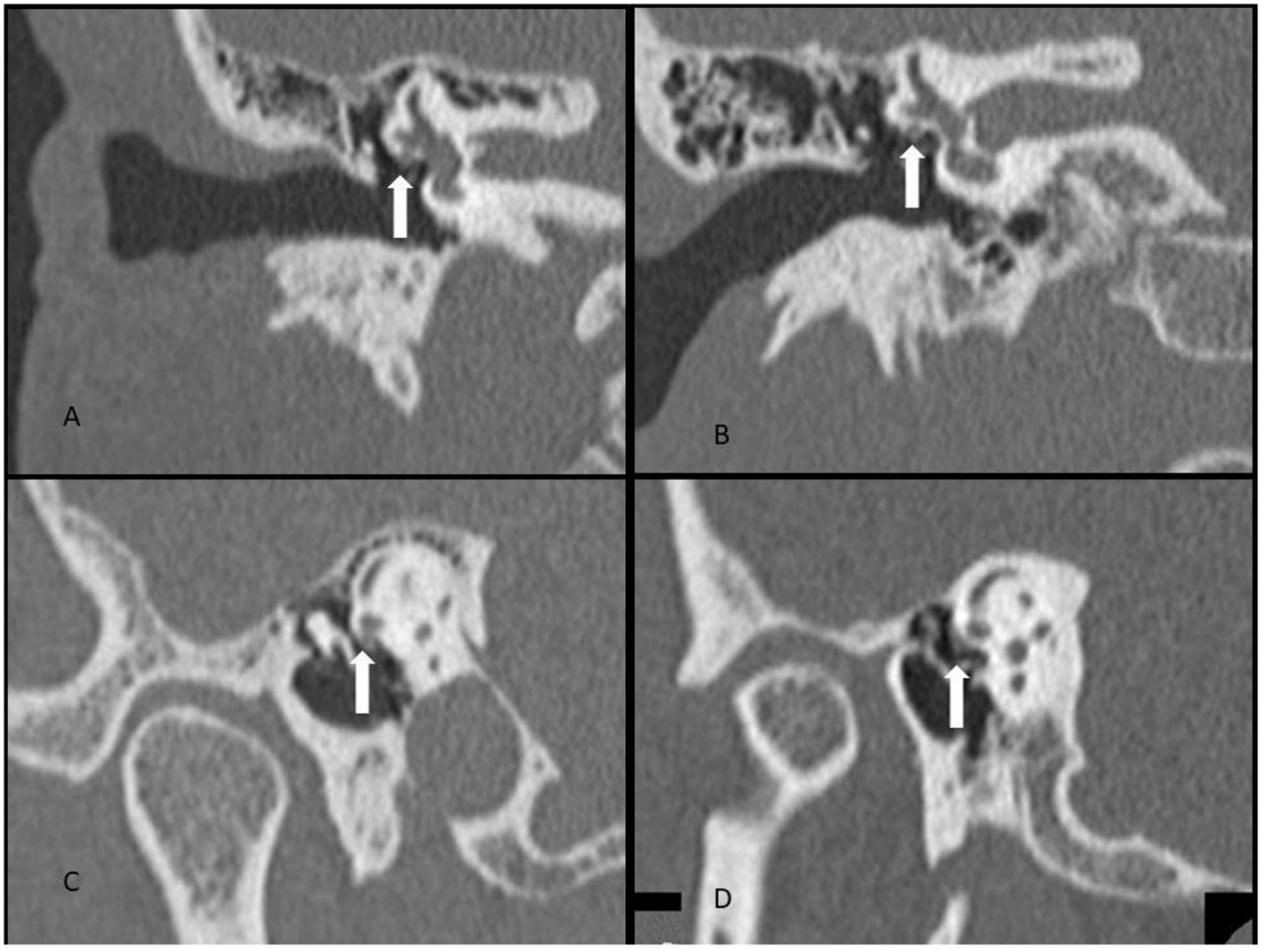
CT scan demonstrating **(A)** HSC-FND on coronal imaging, **(B)** normal HSC and facial nerve anatomy on coronal imaging, **(C)** HSC-FND on Poschl imaging, and **(D)** normal HSC and facial canal on Poschl imaging.

Fallopian canal dehiscence was not systematically recorded, but it seems that all patients had fallopian canal dehiscence noted at surgery. We believe that fallopian canal dehiscence is a necessary part of the anatomy for HSC-FND to become “active.” As noted in Figure 1, there are concomitant defects of the HSC and the fallopian canal at the Oval Window. The concept being the horizontal canal defect leads to pressure changes or an intermittent leak around the facial nerve. If there was no fallopian canal dehiscence, then there would be no means for fluid/pressure movement.

Our treatment paradigm for these patients with HSC-FND has been to begin medical management on all patients when feasible. This resulted in high symptoms resolution rate (71%). For many patients, understanding that straining was provoking many of their vestibular symptoms was revelatory. Educating the patients on how to avoid such provocations resulted in greatly improved quality of life. Further, additional medical measures including salt restriction, diuretics and titration dosing of acetazolamide lowered intracranial pressure and eventually allowed many of the medically treated patients to gradually increase their activity levels without return of vestibular symptoms. For those who continued to have persistent disabling symptoms, surgery was offered.

Of the four patients who did not have resolution on medical management, we offered surgical repair. The surgical repair included soft tissue reinforcement of the HSC-FND inclusive of the oval window and included round window reinforcement. This resulted in complete resolution of vestibular symptoms for three of the patients (one unilateral repair and one bilateral repair by us and one unilateral repair by the referring physician). The fourth patient had been scheduled for sequential bilateral repair and has completed one side as of this writing. This patient has noted significant improvement and we are unsure whether the contralateral surgery will be necessary at this time.

Evidence suggests that otic capsule volume is less and subsequent thickness of the bone surrounding the semicircular canals is thinner in SSCD cases compared with control cases. Park et al. (19) compared the thickness of otic capsule bone covering the semicircular canals in patients with SSCD compared to control cases. Not only did they find thinner bony coverage around the superior semicircular canal, but the bony coverage was also significantly thinner around the posterior and horizontal semicircular canals. Given the reduced volume of otic capsule bone and thinner bony coverage of all three semicircular canals, our study's finding of more than half the patients with other dehiscent sites is not surprising. In addition to HSC-FND, nine of the 16 CT scans reviewed demonstrated dehiscences at other locations in the otic capsule. These other sites included superior canal dehiscences, facial cochlear dehiscences and one case of cochlear carotid dehiscence.

The CT scans of our series of HSC-FND do not identify any erosive process such as cholesteatoma as the source for the dehiscence (see Figure 1 for examples of HSC-FND compared to normal CT scans.). The overlying tympanic segment of the facial nerve suggests a probable anatomic defect that may be of congenital or developmental origin, perhaps due to the reduced otic capsule bone volume as noted by Park et al. (19). Among the 16 cases, 10 (63%) reported the onset of their symptoms immediately after trauma. This is consistent with the second event observation seen with many SSCD patients and suggests a similar pathogenic mechanism for the production of symptoms (12). However, the cause for this bony defect and symptoms onset awaits further investigation.

In the case presented, the patient had all three elements of the triad to make the diagnosis of SCD–history, physiologic testing, and CT scan findings of SSCD. The surgical occlusion would seem to have been performed successfully as demonstrated by improvement of the cVEMP threshold, reduction of the superior canal function on vHIT and absence of the superior canal signal on MRI scan. However, the patient's symptoms were unchanged and our test findings of positive fistula test, Tullio test and Valsalva test strongly pointed to an otic capsule dehiscence. While the abnormal test results could be the results of inadequate SSCD plugging in what appears to be a successful SSCD occlusion procedure, it was surprising to see no improvement in symptoms, especially given reduced anterior canal function on vHIT testing. Alternatively, a concomitant otic capsule dehiscence could explain these findings. Surgical reinforcement as we detailed in the methods section (round window reinforcement and oval window reinforcement inclusive of the adjacent facial nerve segment) by the referring surgeon resulted in resolution of the patient's symptoms. The identification of HSC-FND in this patient provides the likely reason for the patient's persistent symptoms after SSCD surgery. When evaluating patients with SSCD (or any other labyrinthine dehiscence) syndrome, it behooves surgeons to scrutinize the CT scan for additional dehiscent sites. Others have also documented patients with more than one site of dehiscence (5). Proactive treatment of these areas could possibly help improve symptom resolution.

Alternative explanations for the symptomatology in our HSC-FND+O group could be due to the other dehiscences noted in these nine patients. However, the seven patients with no other identifiable dehiscences (HSC-FND group) had a nearly identical symptom and testing profile, consistent with labyrinthine dehiscence. This supports that HSC-FND, as a sole finding, can provoke a similar clinical presentation as other labyrinthine dehiscences.

In our series, the HSC-FND was most evident on both the coronal and Poschl view (a projection perpendicular to the long axis of the petrous bone, at an angle of 45 degrees with the sagittal and coronal planes). This orientation allowed visualization of a cross section of the facial nerve and the bony septum with the horizontal semicircular canal. The axial, sagittal, and Stenvers projections did not demonstrate the dehiscence with the same level of confidence.

Currently, multidetector CT (MDCT) is the mainstay of the diagnosis of a semicircular canal dehiscence. The accuracy in the detection of SSCD in one study was between 92.9% utilizing standard axial and coronal planes and 98.8% using planes parallel and perpendicular to the SSC, similar to the technique and imaging planes as used in this study (20). However, Sequeira et al. (21) caution that MDCT tends to overestimate the prevalence of SSCD in comparison with higher resolution CT techniques such as microCT.

The studies of CT detection for SSCD should alert us to the possibility of falsely positive CT scans. Findings of “dehiscence” on CT, even though not a guarantee of actual bony dehiscence is not without merit. As demonstrated by Ward et al. (14), “near” dehiscence can represent a physiologic phenomenon similar to a frank dehiscence. In our case series, we cannot be certain that all of the identified HSC-FND were bony dehiscences. However, given the patients' symptoms and physiologic test findings, we have a raised degree of confidence that the dehiscences were real or at the very least, represent “near” HSC-FND.

Newer imaging techniques such as microCT and flat panel CT (FPCT) should increase our sensitivity and specificity in detection of HSC-FND (22–24). MicroCT scanning is able to achieve much better resolution than conventional clinical computed tomographic scanning, reducing, or eliminating the chance for error, but cannot be performed on patients at this time because of high radiation dosage and long scanning times. FPCT which uses an area detector instead of detector rows, offers improved spatial resolution for complex anatomy such as the temporal bone, but is currently limited by price and accessibility.

The HSC-FND patients were more likely to have symmetric hearing and evidence of bilateral disease compared to the PLF patients. Bilateral disease was found in 81% of the HSC-FND patients compared to 29% of the PLF patients (*p* < 0.05). This may suggest a congenital/developmental etiology for HSC-FND compared to PLF patients. With regards to hearing, only 25% of HSC-FND patients had asymmetric hearing loss compared to 86% of PLF patients (*p* < 0.05).

This paper has obvious weaknesses, most notably the problems with being a retrospective cases series, which are level IV evidence lacking a good control comparison adding to bias. The inclusion criteria was any new pateint during 2019 who had a CT scan that had been noted in the medical record of possibly having an HSC-FND. Patients with third mobile window symptoms may have had their CT scans more heavily scrutinized for otic capsule defects than those who did not have third mobile window findings. This could potentially bias the selection process and we don't know how many patients with no third mobile window symptoms may have had HSC-FND. However, this could be analogous to finding an asymptomatic SSCD patient. The area in question–the facial nerve as it crosses over the horizontal semicircular canal–is an area where bone is normally thin and could be prone to overdiagnosis as has been seen in CT surveys of SSCD. Further, it is entirely possible that the HSC-FND is not the source of the third mobile window, but another area that has yet been identified could be the source of pathology. These results should be applied cautiously to patients with the above-mentioned clinical profile while carefully scrutinizing for other pathology. As mentioned earlier, the diagnosis of labyrinthine dehiscence requires three elements: (1) symptoms consistent with dehiscence, (2) physiologic test findings consistent with dehiscence and (3) radiographic confirmation of anatomic bony dehiscence. While this study supplies a patient population with all three of these elements, to confirm HSC-FND as a diagnostic entity, histologic studies should be performed to further detail this anatomic area of thin or dehiscent bone.

## Conclusions

A newly described labyrinthine dehiscence is reported. This case series of 16 patients presented with symptoms suggestive of labyrinthine dehiscence–autophony, strain-induced vertigo and Tullio's phenomenon. A history of trauma that immediately preceded the onset of symptoms was seen in most patients. Test findings objectively confirmed the patients' reported symptoms–most commonly by Valsalva testing and less often by fistula testing, Tullio testing and Platform Pressure testing. Imaging with CT demonstrated absence of bone at the ampullated end of the horizontal semicircular canal at the tympanic segment of the facial nerve adjacent to the oval window niche. While nine of these patients had additional dehiscent sites (HSC-FND + O group), seven did not (HSC-FND group). Comparison of clinical symptoms and test findings were not materially different in these two groups, strongly suggesting a similar pathophysiologic process. Compared to a control group of surgical repaired PLF patients, the HSC-FND patients were more likely to have autophony, aural fullness, pulsion sensation, abnormal cVEMP, bilateral disease and symmetric hearing. Medical treatment was successful in symptom resolution in most patients. Surgical reinforcement was successful in the few cases that did not respond to medical management. We recommend consideration of this entity when evaluating patients with symptoms and test findings consistent with labyrinthine dehiscence. We also recommend further confirmation of HSC-FND with temporal bone histologic studies.

## Data Availability Statement

The raw data supporting the conclusions of this article will be made available by the authors, without undue reservation.

## Ethics Statement

The studies involving human participants were reviewed and approved by the Salus Institutional Review Board. Written informed consent for participation was not required for this study in accordance with the national legislation and the institutional requirements.

## Author Contributions

GG, JS, and BS contributed to the data analysis and writing of the paper. All authors contributed to the article and approved the submitted version.

## Conflict of Interest

The authors declare that the research was conducted in the absence of any commercial or financial relationships that could be construed as a potential conflict of interest.

## Publisher's Note

All claims expressed in this article are solely those of the authors and do not necessarily represent those of their affiliated organizations, or those of the publisher, the editors and the reviewers. Any product that may be evaluated in this article, or claim that may be made by its manufacturer, is not guaranteed or endorsed by the publisher.
